# Evaluating tramway infrastructure on biodiversity and ecosystem services

**DOI:** 10.1038/s41598-024-59460-2

**Published:** 2024-04-24

**Authors:** Dawid Moroń, Michał Beim, Agnieszka Gudowska, Fabio Angeoletto, Waldemar Celary, Aleksandra Cwajna, Piotr Indykiewicz, Magdalena Lenda, Emilia Marjańska, Annette Menzel, Piotr Skórka, Piotr Tryjanowski

**Affiliations:** 1 Institute of Systematics and Evolution of Animals, Polish Academy of Sciences, Sławkowska 17, 31–016 Kraków, Poland; 2 Institute of Land Improvement, Environmental Development and Spatial Planning, Poznań University of Life Sciences, Piątkowska 94, 60-649 Poznań, Poland; 3 Programa de Pós-Graduação em Gestão e Technologia Ambiental da UFR, Avenida dos Estudantes 5055, 78735-901 Rondonópolis, MT Brazil; 4 Institute of Biology, The Jan Kochanowski University, Uniwersytecka 7, 25-406 Kielce, Poland; 5 Department of Biology and Animal Environment, Bydgoszcz University of Sciences and Technology, Mazowiecka 28, 85084 Bydgoszcz, Poland; 6 Institute of Nature Conservation, Polish Academy of Sciences, Adama Mickiewicza 33, 31-120 Kraków, Poland; 7 TUM School of Life Sciences, Ecoclimatology, Technical University of Munich, 85354 Freising, Germany; 8 Institute for Advanced Study, Technical University of Munich, 85748 Garching, Germany; 9 Department of Zoology, Poznań University of Life Sciences, Wojska Polskiego 71C, 60-625 Poznań, Poland

**Keywords:** Ecosystem services, Green urban architecture, Human-nature conflict reduction, Land sharing, Transport infrastructure, Urban planning, Biodiversity, Conservation biology, Ecosystem services, Urban ecology

## Abstract

Tramways in urban areas for mass transit has been suggested to have a lower environmental footprint than roads. However, studies on the impact of tramways and the surrounding infrastructure on biodiversity is extremely rare despite the potential ecological effects associated with this anthropogenic feature. Surprisingly, we found fewer than 10 papers published on tramway-wildlife interactions, which is significantly lower (*vs* dozens of thousands) than that of other transportation methods. As tramways and stations may be managed sustainably by planting short vegetation on the track and roofs of tramway stations, they may be good examples of land-sharing policies in green urban planning, improving both biodiversity and people’s well-being. The potential environmental benefits of green practices for commercially available tramways should be strictly tested and applied, especially in the context of the growing popularity of tramway systems worldwide.

## Introduction

Human car accidents due to collisions with animals and road mortality are main causes of human–nature conflicts and may lead to fewer green urban environments being planned^1^. In the context of traffic, human-wildlife conflicts can be defined as encounters between humans and wildlife, resulting in negative outcomes for both humans and their resources, and wildlife and their habitats^2^. Generally, roads and railways are recognised as linear landscape structures that negatively impact several species^3^. For example, tens of millions of birds are killed annually owing to collisions with automobiles in the US^4^, billions of pollinating insects are killed per annum across North America^5^, and hundreds of ungulates are subject to yearly railroad collisions^6^. However, it has been demonstrated that linear landscape structures, such as roads and railways, can positively benefit some species by providing foraging and nesting possibilities or pose migratory routes^7,8^, as well as benefit non-native species^9^. Associated linear landscape structures such as power lines or fences, can be used by insects as nesting spots^10,11^, by plants to climb up structures^12^, and by birds as perches for hunting activities, singing and displaying, or simply for resting^13,14^. Moreover, some bird species use railways to clean feathers during sand-bathing or collect grit as a source of calcium and as gastroliths^13^. However, the impact of transportation on the environment, especially biodiversity, is limited to roads and some aspects of railways^15,16^.

However, in urban areas of many regions of the world, trams are a very popular type of public transport, sometimes in historical contexts recognised as streetcars in the USA or modern light rail transit/light railway vehicles^17^. Tramways are intensively developed, and some aspects such as availability of stops points, design, speed, and low impact on the environment are especially important to passengers and inhabitants^18^. Tramway networks in cities have infrastructure similar to roads, such as paved surfaces, drainage, bridges, poles, or stops, and may provide similar pitfalls and opportunities for wildlife across different continents, countries, or cities^19^. Unfortunately, little is known about how these elements affect urban populations of animals and plants. Trams, a form of mass public transport, have been suggested environmentally friendly. However, surprisingly, tramways have not been studied in detail^3,20, 21^. Analysis of the potential influence of this type of public transport on wildlife seems to be important, especially in light of the resurrection and extension of tramway networks in many European cities in the last decade^18^ and significant new investments, such as NextGenerationEU (https://ec.europa.eu/info/strategy/recovery-plan-europe_en). On the other hand, since species may affect transport safety through collisions^22^, as well as by other means, for example, metal corrosion caused by excreta^23^, knowledge on how wildlife may use the transport infrastructure is important not only for biodiversity but also for ecosystem services, for example human safety purposes, cooling effects, and water retention. Thus, monetary methods for quantifying the non-market benefits from greening tramways and estimating increases in their ecosystem service values should be applied for sustainable decision-making in urban areas^24,25^.

The renaissance of trams originated in France, where a school for designing modern tram systems was developed. In addition to an innovative approach to urban issues (e.g. tram routes), the French School of Design is characterised by the widespread use of green tracks^26^. However, as historical photographs, films, and postcards show, green tracks have existed since the second decade of the twentieth century. The first city to turn green tracks into a symbol of a modern means of transport to protect the environment, and as a symbol of the city at all, was Freiburg im Breisgau. In 1978, a new line to Landwasser was opened. The new investment was characterised by several innovations, including green tracks^27^. Originally, green tracks were expected to improve aesthetics, reduce noise spread, and cool the urban heat island, in comparison to traditional tracks on ballast. With the popularity of green tracks, different types (grass track, sedum track, high- or low-level, etc.) and construction techniques have been distinguished^28^. Accordingly, green tracks are increasingly becoming a subject of local public policies, e.g. Urban Heat Island Strategy City of Vienna^29^; a subject of research projects, e.g. German-wide Grüngleisnetzwerk^28^; or a part of an Urbact project “RiConnect—Rethinking infrastructure” (https://urbact.eu/networks/riconnect).

It is likely that interest in public transport, after the temporary stagnation resulting from the COVID-19 lockdown^30^, will systematically increase. Therefore, it is important to prepare for a long-lasting debate on its importance, possibilities, limitations, and environmental impact in a changing world^31^. Importantly, management schemes based on policy-focused analysis should be ready for business and government administrations for world rebuilding after large-scale disturbances such as pandemics or climate change. Recently, ideas such as land-sharing and land-sparing have been woven into urban ecology, aiming to harmoniously blend green spaces with economic activities in cities^32,33^. Land-sharing advocates gently interspersing urban development with green elements, such as trees, grass, or small parks, nestled among structures. Green tramways may be a good example of land sharing policy in urban development using linear landscape structures, reducing human–nature conflict by combining active management and using the area for public transportation in urban areas, providing biodiversity, and benefiting human wellbeing^34^.

Therefore, there were two main aims of our study: (1) to summarise the state-of-the-art ways in which tramways and surrounding infrastructure affect biodiversity underlying commonness in urban landscapes worldwide, as well as the importance of trams for societies; and (2) to analyse potential gaps in the knowledge of the importance of trams for biodiversity, including wildlife. To realise the above purposes, in this study, we collected and classified available information on the main effects of trams and associated infrastructure on biodiversity. We hope to provide useful records for ecologists, road planners and other stakeholders engaged in conservation and urban planning.

## Methods

### Systematic review

A search of the relevant peer-reviewed literature was conducted using the Web of Science and Scopus databases on 26 January 2023. A set of keywords was used in the following search string: (tramway* OR trams OR “tram* track*” OR streetcar* OR “light rail transit” OR “light railway vehicles” OR lrt OR lrv) AND (*diversity OR wildlife OR vegetation* OR flora OR fauna OR richness OR disturbance OR birds OR mammals OR amphibians OR reptiles OR insects ) AND NOT (“Tram Chim”). The search was limited to the subject areas of Environmental Science, Agricultural and Biological Sciences in Scopus and was refined by the following Web of Science categories: Environmental Sciences, Ecology, Environmental Studies, Multidisciplinary Sciences, Plant Sciences, Evolutionary Biology, Biodiversity Conservation, Engineering Environmental, Biology, Horticulture, Ornithology, Zoology. Using the above search method, we identified 108 articles from Scopus and 82 from the Web of Science. After removing duplicates, 136 unique entries were considered for abstract screening. Based on the title, abstract, and keywords, we screened in Rayyan QCRI (https://rayyan.qcri.org/). We also included one additional record identified by a backward search of the previously included studies. We found only eight empirical studies published between 2013 and 2022 that investigated the impact of tramways on wildlife (Table 1). The remaining 128 papers subjected to screening were irrelevant regardless of the usage of specified keywords in the search strings.

**Table 1:** Studies included in this review.

City	Group	Species	The impact of trams	References
Poznań (Poland)	Birds	Jackdaw *Corvus monedula*	The tram infrastructure is widely used by urban bird species, mainly as foraging and resting places. Tram tracks appear to be safe foraging places for birds, especially for corvids.	^42^
		Feral pigeon *Columba livia*		
		Rook *Corvus frugilegus*		
		Magpie *Pica pica*		
		Hooded crow *Corvus cornix*		
		Starling *Sturnus vulgaris*		
		Blackbird *Turdus merula*		
		Fieldfare *Turdus pilaris*		
		Eurasian collared dove *Streptopelia decaocto*		
		Common wood pigeon *Columba palumbus*		
		Eurasian jay *Garrulus glandarius*		
Poznań (Poland)	Birds	Hooded crow *Corvus cornix*	Each investigated species showed selectivity for a different set of habitat features. The abundance of hooded crows was positively influenced by the length of tram tracks.	^61^
		Rook *Corvus frugilegus*		
		Jackdaw *Corvus monedula*		
		Magpie *Pica pica*		
		Jay *Garrulus glandarius*		
Melbourne (Australia)	Birds	Feral pigeon *Columba livia domestica*	Silver gulls were present in large numbers only in areas with the least disturbance from traffic and trams (and their overhead wires).	^43^
		House sparrow *Passer domesticus*		
		Silver gull *Chroicocephalus novaehollandiae*		
		Common myna *Sturnus tristis*		
		Spotted dove *Streptopelia chinensis*		
		Little corella *Cacatua sanguinea*		
Poznań (Poland)	Newts	Smooth newt *Lissotriton vulgaris*	Observed mortality was very low (less than 1% of all individuals found during the survey) despite the large number of individuals present on the track and intensive tram traffic. As negative effects of traffic are low, rail or tram embankments can provide an important terrestrial habitat for small European newts.	^21^
Bratislava (Slovakia)	Plants	The most frequent taxa (see full list in the original paper):	Significant differences in flora found strictly within the rail yard and those growing at a greater distance from the tracks (i.e. tracksides). The number of alien species recorded directly in the rail yard was higher than on the tracksides.	^39^
		*Achillea millefolium* agg.		
		*Cichorium intybus*		
		*Eragrostis minor*		
		*Plantago lanceolate*		
		*Polygonum arenastrum*		
		*Portulaca oleracea*		
		*Taraxacum* sect.		
		*Ruderalia*		
Bratislava (Slovakia)	Plants	123 taxa spontaneously growing on the strict rail yard of Bratislava tram tracks and 96 taxa spontaneously growing on tracksides.	Significant differences in the composition of flora between conventional tram tracks and green tram tracks. Green tram tracks host fewer spontaneously growing taxa than conventional ones. Both in older, conventional tram tracks and newer green tram tracks archaeophytes were more abundant than neophytes, however, they also host a relatively high proportion of alien species.	^40^
Alexandria (Egypt)	Plants	224 species were recorder in the study.	Tram tracks maintaining higher vitality and cover compared to train tracks. Species recorded were mainly therophytes, followed by phanerophytes and hemicryptophytes dominated by native species; however, invasive species’ contribution was higher compared to surrounding regions. The number of invasive species was greater in railway areas compared to tram track areas. These habitats are valuable refuge areas for rare and endangered species worthy of conservation action.	^38^
Szczecin (Poland)	Plants	421 taxa.	The area associated with trams developed a mosaic habitat with the specific spontaneous and relatively rich flora. Plant composition is the result of adaptation to extreme human pressure on the habitat. The profile of the flora of the tramway areas is similar to that of the flora of industrial or urban habitats. Additionally, six protected species, as well as two rare and endangered plants were found.	^41^

### Global interest

We used Web of Science database (https://www.webofscience.com/wos/woscc/basic-search) to assess the changes in tramway studies published over time. In March 2023, a literature search was conducted using the Web of Science Core Collection for papers published between 1950 and 2022 that included the term “tramway” in their titles.

To assess public interest in trams as transport over time, we used the Google Trends database (https://trends.google.com/trends/). Google Trends is a public web facility provided by Google Inc. that measures how often a particular search item is entered into Google Search browsers relative to the total search volume. The trends provided by this tool estimate changes in searches for an item or phrase and are often used to examine temporal changes in socio-economic studies^35^. The search for the term “tram” was performed on 7.03.2023 and the region was set to World. To avoid biases, we set the “travels” filter, allowing searches to only find travel-related items when searching for trams as transport, thereby avoiding searches for other purposes.

### Trams all over the World

The lengths of the tramways and light rail transit networks of cities, as well as the populations of cities, were obtained from Wikipedia. Wikipedia is a multilingual, free online encyclopaedia written and maintained by a community of volunteers (https://en.wikipedia.org/wiki/Wikipedia). Cities listed in the Wikipedia page “List of tram and light rail transit systems” (https://en.wikipedia.org/wiki/List_of_tram_and_light_rail_transit_systems) along with information about their tramway network (in English) were included in the database. In most cases, the length of a city tramway network is referred to as the length of the lines, routes, systems, or tracks. The data include networks which provide actual transit services (including heritage trams and streetcars), not those that are presently under construction or are qualified as metro networks. Networks in Russia and Turkey, including those in the European regions, are listed for convenience under Asia.

### Data visualization

All visualizations were performed with R^36^, package ggplot2^37^.

## Results

### Systematic review

Although tramways are frequent elements of many urban landscapes in the EU and other countries, their contribution to city biodiversity has not been thoroughly studied (Table 1). We found eight empirical studies published between 2013 and 2022 that investigated the impact of tramways on wildlife (Table 1). It is already recognized that tramways result in the development of a mosaic habitat covered by many plant species, including both spontaneous flora and cultivated plants^38–41^ (Table 1). The floral composition profile of infrastructure associated with tram communication is similar to that of the flora of industrial or urban habitats^41^. The recorded plant species were dominated by native species, but the tramways were also sources of alien and invasive taxa. However, the potential risk of plant invasion differs among the tramway infrastructure types. Rendeková et al.^40^ revealed that green tramways are habitats with fewer spontaneously growing alien taxa, and their frequency of occurrence is lower than that on conventional tracks. In the case of conventional tracks, alien species occurred directly in the rail yard more frequently than those growing at greater distances from the tracks^39^. Despite the abovementioned risks at these sites, tramways can be a valuable refuge for endemic and endangered species worthy of conservation action^38,39^. Moreover, city wildlife seem to use tramways as attractive habitats for food foraging, resting, or moving along. However, to the best of our knowledge, the value of tramways for animals has only been studied for birds and newts (Table 1). Szala et al.^42^. showed that the tramway infrastructure is used by 11 bird species, particularly corvids and pigeons. In winter, the abundance of hooded crows was positively influenced by tramway length. These habitats may constitute valuable foraging areas, especially during severe winter^42^. In contrast, some birds, such as silver gulls (*Chroicocephalus novaehollandiae*), avoid areas with high disturbance from traffic, trams, and overhead wires, despite the high availability of food at these sites^43^. Furthermore, tramways are important terrestrial habitats for smooth newts in late autumn and winter (*Lissotriton vulgaris*^21^). The rail aggregate provides a large number of shelters and cavities, thus reducing predation risk, and providing a prey-rich, humid habitat. Moreover, dense tramway networks may encourage more people to use tramways instead of cars, further reducing animal road mortality and pollution.

### Global interest

**Figure 1:**
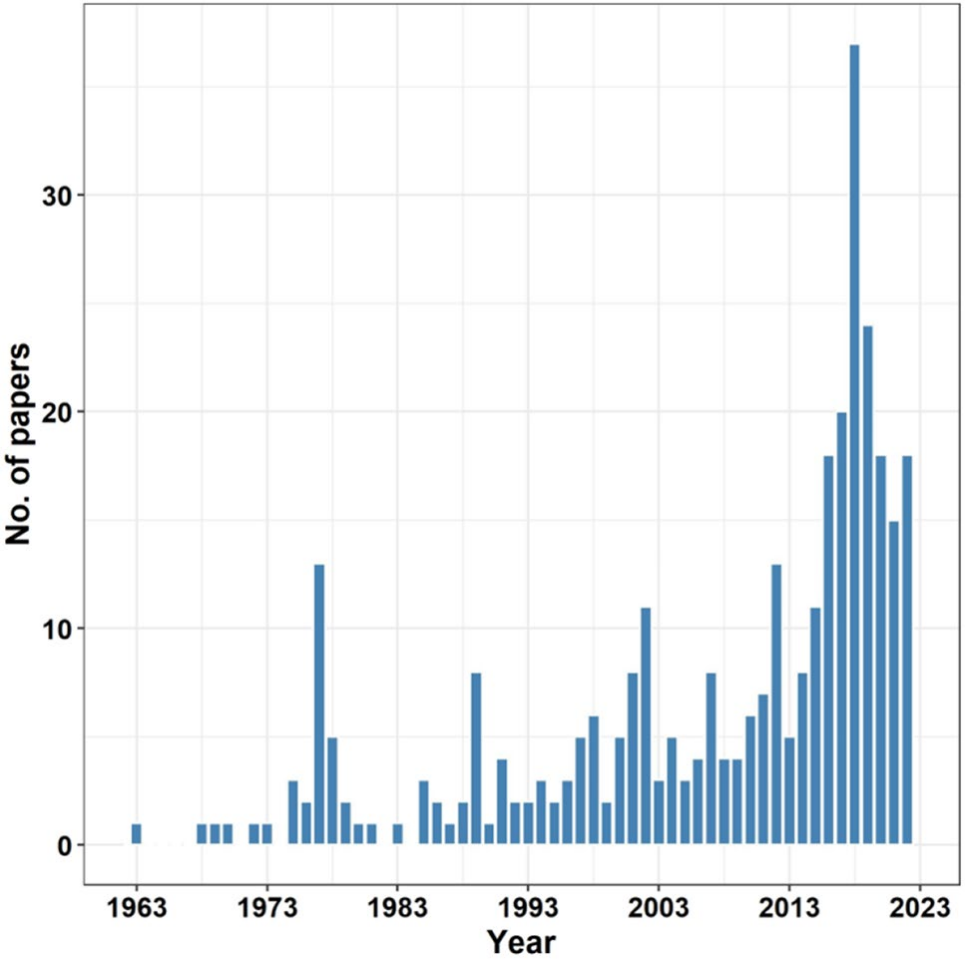
The total number of published papers with the term “tramway” in a title. The literature search was conducted using Web of Science.

Among the 335 studies containing the term “tramways” in their titles published between 1955 and 2022 (Fig. 1), the Web of Science Categories matched were: Transportation Science Technology (19%), Engineering Civil (13%) and Engineering Electrical Electronics (13%). The number of publications increased over time with a peak observed in the year 2017.

**Figure 2:**
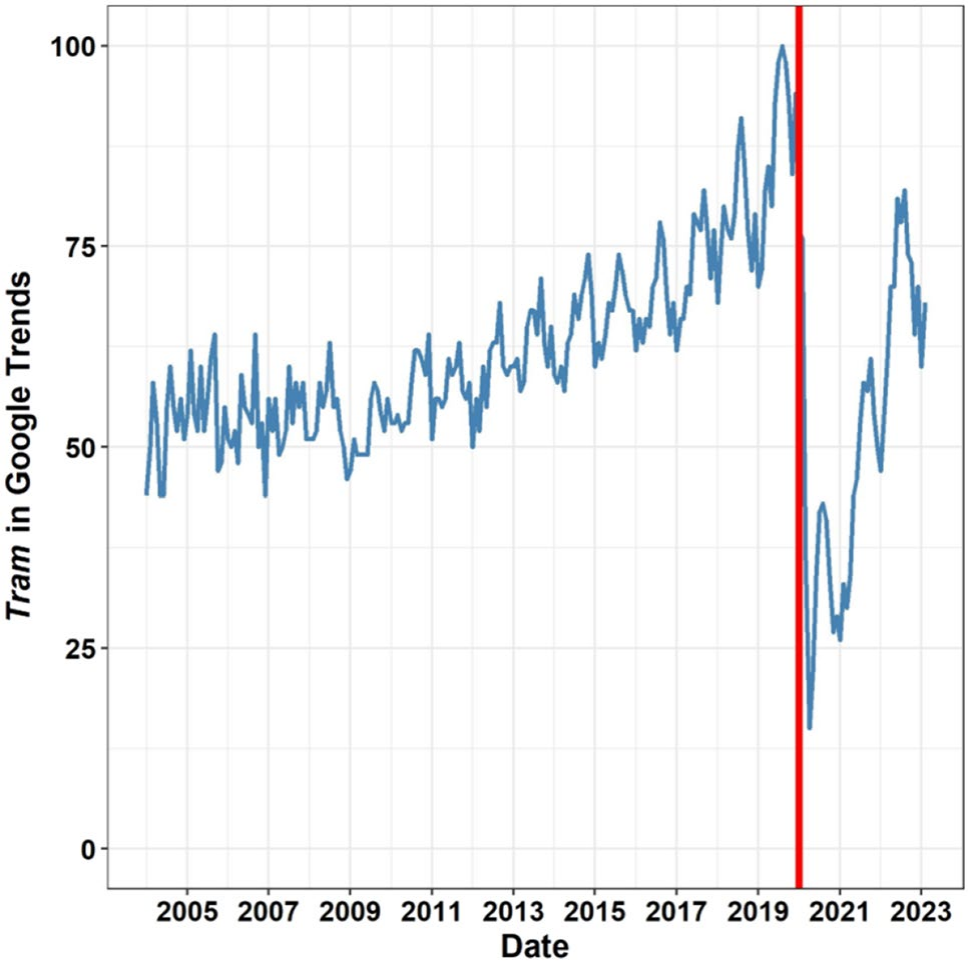
The blue line represents Annual Google Trend searches for the term “tram”. The red line marks the beginning of the COVID-19 pandemic, when public transport was restricted. The search was conducted across the entire world and the “travels” filter was set.

Moreover, the trend of the Internet search for the term “tram” increased from the year 2005, but significantly decreased during the COVID-19 pandemic (Fig. 2).

### Trams all over the World

**Figure 3:**
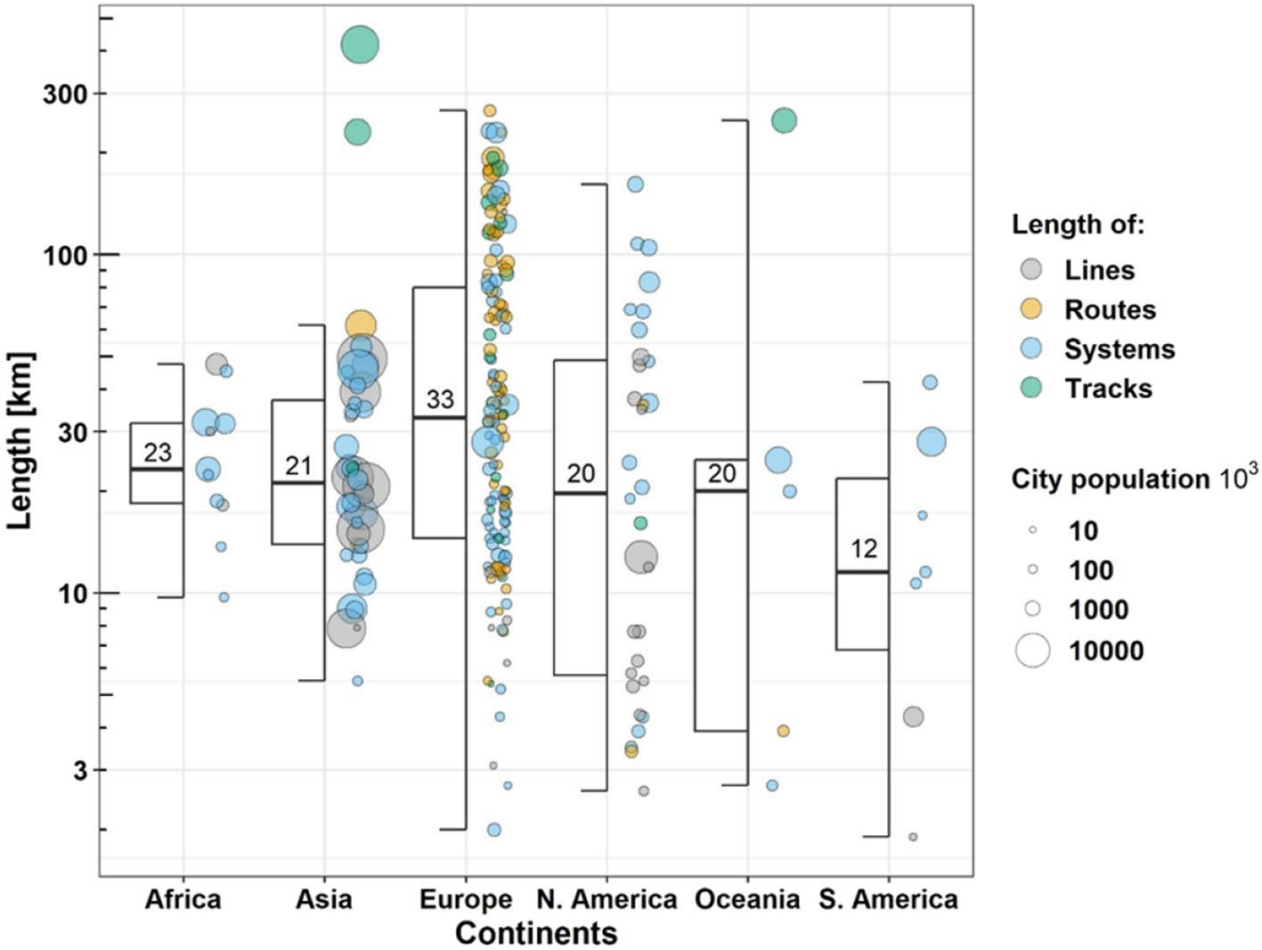
Length of tramway and light rail transit network of cities in six continents. Colour of points correspond with the referred length of a city’s tramway lines, routes, systems or tracks. Circles are arranged according to population size of a city (in thousands). The median length is presented as values in boxes. Jittering was added to aid visualization.

The median value of the tramway network length was highest for European cities (33 km) and lowest for South American cities (12 km; Fig. 3). The tramway network lengths for Africa, Asia, North America and Oceania ranged between 23 and 20 km (Fig. 3).

## Discussion

Significant efforts have been made to develop of protection plans to recover or sustain the current level of biodiversity and ecosystem services in urban areas^44^. Interventions in urban landscapes encouraging landowners to properly design gardens or create wildlife sanctuaries have been devised with the hope that wildlife will survive^45,46^. However, this approach to conserving species diversity faces many practical problems. The effectiveness of wildlife sanctuaries in an urban landscape depends on where they are implemented, the genus or order of the plants and animals being targeted, and the landscape structure^31,44^. Sanctuaries may be located in areas isolated from other semi-natural habitats and might play a minor role as a source habitat^47^. Many solutions for wildlife in urban areas are costly, and hence may be limited to the local scale or well-developed countries^48^.

A supplementary or alternative solution to the above-mentioned methods is to take advantage of the unrecognised benefits of artificial or novel habitats for wildlife^49^, according to the land-sharing concept^33^. Such novel habitats, usually associated with industrial or infrastructural development, may have high conservation value. For example, it has been shown that limestone quarries^50^, road verges^51^, former open-surface coal mines^52^, landfills^53^, sandpits^54^, gravel-pits^55^, gardens^45^, railway embankments^56^, levees^57^, or green roofs^46^ may be refuges for pollinator populations. Moreover, linear landscape structures, such as railways or levees, may act as corridors for insects that are highly affected by human landscape^8^. Thus, habitats created by human activities may significantly mitigate the negative effects of industry and agriculture^58^. Tramways are common landscape features worldwide (Fig. 3) which increase their potential value for biodiversity conservation and restoration of ecosystem services. Additionally, steadily increasing the interest of society and scientists in trams (Figs. 1,2), e.g. in an era of transportation rethinking, may lead to favourable conditions for implementing new ideas of bringing biodiversity back to cities^59^.

**Figure 4:**
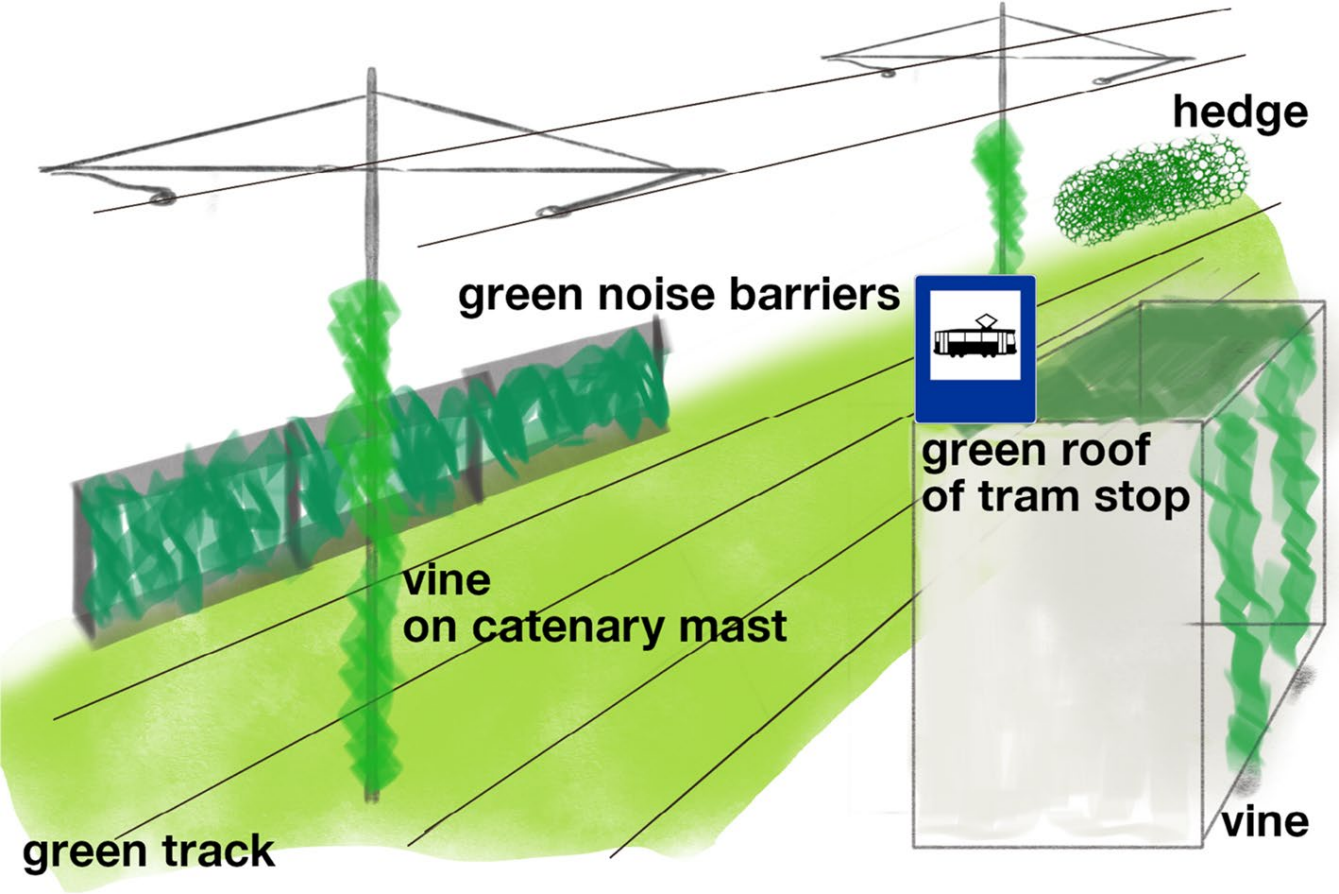
Exemplary greening of tram infrastructure.

Having recognised the positive aspects of tramways for wildlife, one should also be aware of the possible threats to biodiversity brought about by tramways^60^. Tram traffic can cause animal mortality, and thus lower population abundance. However, there is no strong evidence suggesting that tram traffic kills many animals. Surprisingly, for both birds and newts, tramway infrastructure does not seem to be dangerous, and it is not an additional source of mortality, in contrast to roads^21,61^, perhaps because of the average speeds of trams and cars. Tram traffic, even in the busiest lines, is much lower than the traffic volume on roads, thus, it is reasonable to assume that mortality is probably lower than that on roads. Tramway transport can also be a serious source of different kind of pollutions^62,63^ which may negatively impact wildlife. Pollution also includes non-selective herbicides used to maintain tracks^64^ which, in turn, may negatively affect insect populations^65^, e.g. by lowering native flowering plant cover. Although tramway verges may act as functional biological corridors^8^, these may also pose a barrier for wildlife. Movements between habitat patches may also be diminished by tramways that “filter” individuals who are unwilling to move further when they encounter the tracks^66^. However, this indicates that the potential role of tramway infrastructure is even greater and that applying alternative methods of vegetation management may increase the positive role of this habitat (Fig. 4). Additionally, moving trams may also be a source of noise pollution; however, modern tracks and trams typically exhibit reduced noise emissions.

### Management recommendations

**Figure 5:**
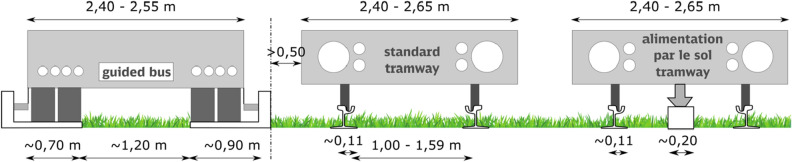
Space for greenery in tracks of guided buses, typical tramways and tramways supplied with electric power by the conductor rail built into the track (*l'alimentation électrique par le sol*).

**Figure 6:**
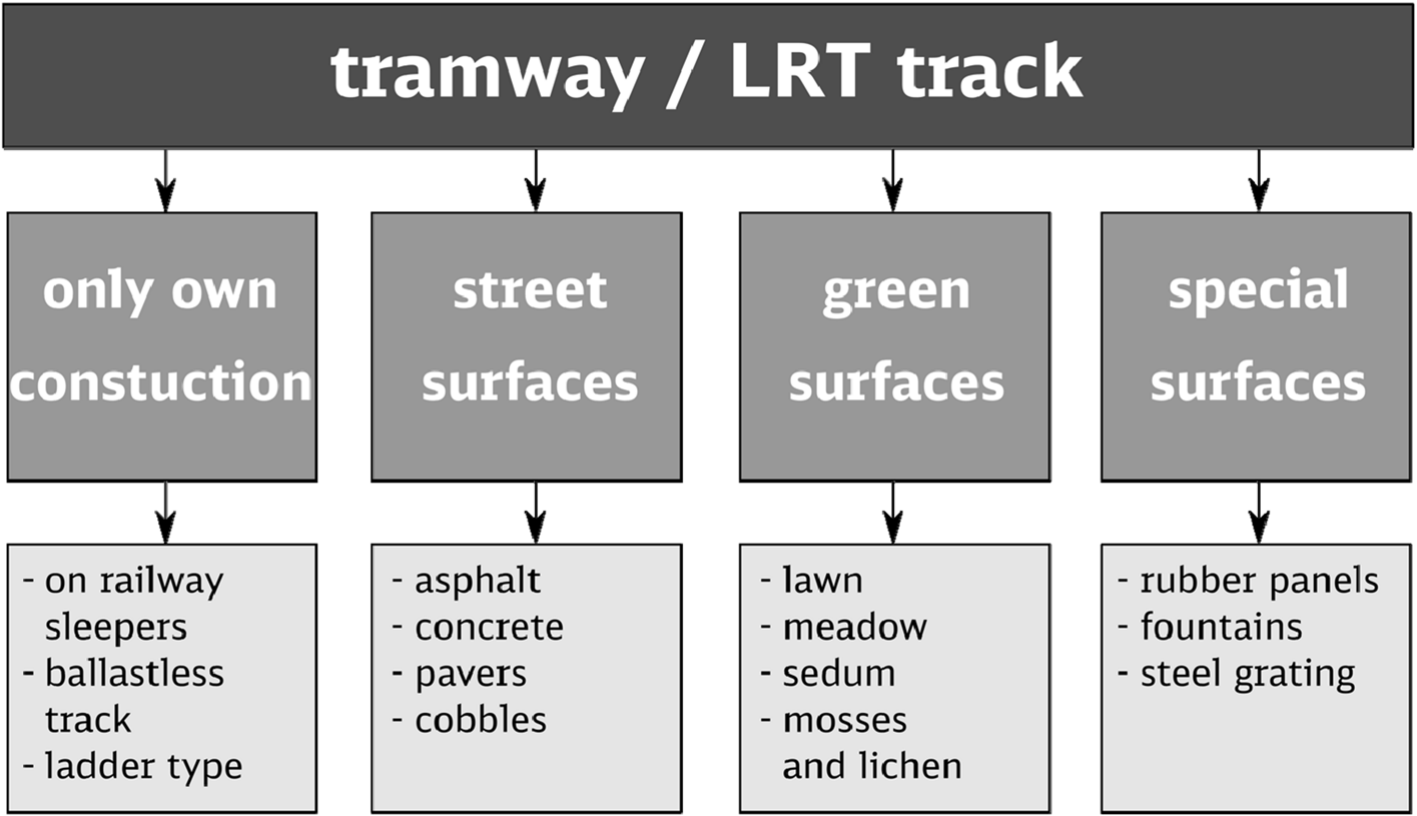
Types of location of tracks in the urban space.

Tramway systems have evolved with technological advancements. Analysing public, political, and scientific debates, one of the key directions of technical development is the use of trams moving without overhead traction (Figs. 5, 6). This is primarily due to the protection of historic areas. There is also an interesting discussion in Munich regarding the use of catenary-free tramways to protect natural and landscape values in the planned northern tramway rings. “Tram-Nordtangente”, is planned to be a 2200 m long double-track line. Approximately 800 m will be located in the English Garden [German: Englischer Garten], one of the most famous parks in the city. This is the most controversial aspect of new investments. The primary consideration is to reduce the environmental impact of a new tramway^67,68^. The removal of overhead lines and equipment from cities was considered in two contexts. The first is the ground-level power supply, which, as mentioned above (Figs. 5,6), reduces the area of the biologically active surface, making it difficult to maintain because it divides the area between the rails into two narrow strips of greenery approximately 60 cm wide. The construction of *l'alimentation électrique par le sol* also requires protection against flooding. The second consideration is the development of battery or supercapacitor systems or the use of hydrogen fuel cells. Although the application of ground-level power supply or battery (supercapacitor) run trams is a common practice worldwide, hydrogen fuel cell trams remain a topic of research and development, e.g. the H2-Tram Project in Germany^69,70^.

It should be mentioned that there were many concerns about the development of green tracks at the turn of the 1980s and the 1990s, when low-floor (in various cities) or very low-floor (e.g. Vienna, Oradea) trams were introduced. However, this has not prevented the development of green tracks.

For sustainable tramway development, measurement of monetary losses of ecosystem services when a tram line is built, or ecosystem services gained after greening the existing lines should be utilized^71^. Methods of nonmarket valuation, such as the Biotope Valuation Method and Energy-Water-Vegetation Method can show the range of environmental values of nature. This includes assessing the societal costs of restoring landscape quality to its real ability to replace the core supporting and regulating services of ecosystems, such as climatising services, water-retention services, oxygen production, and habitats for biodiversity^72^. Thus, the estimated values for ecosystem services per unit length of green tram tracks should be incorporated into decision-making in urban landscapes.

### Future studies

There has been a significant increase in the number of studies on tramway engineering (Fig. 1), social interest in trams as transport networks (Fig. 2) and urban management plans that consider greening tramways^28^. This is not surprising as tramway features are common in urban landscapes on all continents (Fig. 3). However, there is an urgent need to consider the effects, advantages, and disadvantages, of tram transport on biodiversity and ecosystem services in cities. Specifically, we need to understand the potential role of tramway infrastructure in:

- creating new habitats for biodiversity of rare/key group species,

- improving ecological processes, such as migration and primary production,

- increasing the economic valuation of ecosystem services (cooling—climate change, pollination, water retention, and aesthetic values),

- assessing and preventing the mortality of animals through engineering solutions,

- incorporating tramways into urban development strategies, that is, land sharing versus land sparing.

## Data Availability

All data supporting the findings of this study are available in the manuscript (figures, tables, and references).

## References

[1] Bryant MM (2006). Urban landscape conservation and the role of ecological greenways at local and metropolitan scales. Landsc. Urban Plan..

[2] Schell CJ (2021). The evolutionary consequences of human–wildlife conflict in cities. Evol. Appl..

[3] van der Ree, R., Smith, D. J. & Grilo, C. *Handbook of Road Ecology*. (John Wiley & Sons, 2015).

[4] Loss SR, Will T, Marra PP (2014). Estimation of bird-vehicle collision mortality on U.S. roads. J. Wildl. Manag..

[5] Baxter-Gilbert JH, Riley JL, Neufeld CJH, Litzgus JD, Lesbarrères D (2015). Road mortality potentially responsible for billions of pollinating insect deaths annually. J. Insect Conserv..

[6] Jasińska KD (2019). Linking habitat composition, local population densities and traffic characteristics to spatial patterns of ungulate-train collisions. J. Appl. Ecol..

[7] Phillips BB, Gaston KJ, Bullock JM, Osborne JL (2019). Road verges support pollinators in agricultural landscapes, but are diminished by heavy traffic and summer cutting. J. Appl. Ecol..

[8] Moroń D, Skórka P, Lenda M, Celary W, Tryjanowski P (2017). Railway lines affect spatial turnover of pollinator communities in an agricultural landscape. Divers. Distrib..

[9] Lemke A, Buchholz S, Kowarik I, Starfinger U, von der Lippe M (2021). Interaction of traffic intensity and habitat features shape invasion dynamics of an invasive alien species (*Ambrosia artemisiifolia*) in a regional road network. NeoBiota.

[10] Sobieraj-Betlińska A, Szefer P, Twerd L (2023). Linear woodlots increase wild bee abundance by providing additional food sources in an agricultural landscape. Agric. For. Entomol..

[11] Twerd L, Sobieraj-Betlińska A, Szefer P (2021). Roads, railways, and power lines: Are they crucial for bees in urban woodlands?. Urban For. Urban Green..

[12] Benoliel MA, Manso M, Ferreira PD, Silva CM, Cruz CO (2021). “Greening” and comfort conditions in transport infrastructure systems: Understanding users’ preferences. Build. Environ..

[13] Morelli F, Beim M, Jerzak L, Jones D, Tryjanowski P (2014). Can roads, railways and related structures have positive effects on birds?—A review. Transp. Res. Part D Transp. Environ..

[14] Tryjanowski P, Sparks TH, Jerzak L, Rosin ZM, Skórka P (2014). A paradox for conservation: Electricity pylons may benefit avian diversity in intensive farmland. Conserv. Lett..

[15] Torres A, Jaeger JAG, Alonso JC (2016). Assessing large-scale wildlife responses to human infrastructure development. Proc. Natl. Acad. Sci..

[16] Popp JN, Boyle SP (2017). Railway ecology: Underrepresented in science?. Basic Appl. Ecol..

[17] Jones P (1978). Innovation life-span: The urban tramway. Area.

[18] Hickman R, Hall P, Banister D (2013). Planning more for sustainable mobility. J. Transp. Geogr..

[19] Barrientos R, Ascensão F, Beja P, Pereira HM, Borda-de-Água L (2019). Railway ecology vs. road ecology: Similarities and differences. Eur. J. Wildl. Res..

[20] Borda-de-Água, L., Barrientos, R., Beja, P. & Pereira, H. M. *Railway Ecology*. (Springer International Publishing, 2017).

[21] Kaczmarski M, Kaczmarek JM (2016). Heavy traffic, low mortality—Tram tracks as terrestrial habitat of newts. Acta Herpetol..

[22] Gunson KE, Mountrakis G, Quackenbush LJ (2011). Spatial wildlife-vehicle collision models: A review of current work and its application to transportation mitigation projects. J. Environ. Manag..

[23] Spennemann DHR, Watson MJ (2018). Experimental studies on the impact of bird excreta on architectural metals. APT Bull. J. Preserv. Technol..

[24] Seják J, Pokorný J, Seeley K, Skene KR (2022). Why ecosystem services should be counterbalanced by nature’s thermodynamic costs. Ecosyst. Serv..

[25] Costanza R (1997). The value of the world’s ecosystem services and natural capital. Nature.

[26] Désveaux, D., Richez, T., Blerot, F. & Cottet, V. *Tramways-à-la-française*. (Archibooks, 2013).

[27] Beim M, Haag M (2014). Public transport as a key factor of urban sustainability. A case study of Freiburg From balanced development to sustainable development. Badania Fizjogr. Ser. D Gospod. Przestrz..

[28] Kappis, C. & Schreiter, H. *Handbook track greening*. (Eurail Press, 2016).

[29] Damyanovic, D. *et al.* Pilot Action City of Vienna—UHI-STRAT Vienna. in *Counteracting Urban Heat Island Effects in a Global Climate Change Scenario* (ed. Musco, F.) 257–280 (Springer Open, 2016).

[30] Dong H, Ma S, Jia N, Tian J (2021). Understanding public transport satisfaction in post COVID-19 pandemic. Transp. Policy.

[31] Łukaszkiewicz J, Fortuna-Antoszkiewicz B, Oleszczuk Ł, Fialová J (2021). The potential of tram networks in the revitalization of the Warsaw Landscape. Land.

[32] Green RE (2005). Farming and the fate of wild nature. Science.

[33] Soga M, Yamaura Y, Koike S, Gaston KJ (2014). Land sharing vs. land sparing: Does the compact city reconcile urban development and biodiversity conservation?. J. Appl. Ecol..

[34] Lenda M (2023). Recognizing the importance of near-home contact with nature for mental well-being based on the COVID-19 lockdown experience. Ecol. Soc..

[35] Lenda M (2021). Misinformation, internet honey trading and beekeepers drive a plant invasion. Ecol. Lett..

[36] R Core Team. *_R: A Language and Environment for Statistical Computing_ R Foundation for Statistical Computing.* (R Foundation for Statistical Computing, 2023).

[37] Wickham, H. *ggplot2: Elegant Graphics for Data Analysis*. (Springer-Verlag, 2016).

[38] Heneidy SZ (2021). Pattern of urban flora in intra-city railway habitats (Alexandria, Egypt): A conservation perspective. Biol. Basel..

[39] Rendeková A (2020). Flora of the tram tracks of Bratislava. Urban Ecosyst..

[40] Rendeková A (2022). Comparison of the differences in the composition of ruderal flora between conventional tram tracks and managed green tram tracks in the urban ecosystem of the city of Bratislava. Hacquetia.

[41] Klera M, Bacieczko W (2013). Specific of the flora of the tramway infrastructure of Szczecin as the manifestation of an extreme synanthropization of biotope. Folia Pomeranae. Univ. Technol. Stetin..

[42] Szala K, Dylewski Ł, Tobolka M (2020). Winter habitat selection of Corvids in an urban ecosystem. Urban Ecosyst..

[43] Pike M, Spennemann DHR, Watson MJ (2017). Building use by urban commensal avifauna in Melbourne central business district Australia. Emu Austral. Ornithol..

[44] Williams NSG, Lundholm J, Scott MacIvor J (2014). Do green roofs help urban biodiversity conservation?. J. Appl. Ecol..

[45] Plummer KE (2024). Trends in butterfly populations in UK gardens—New evidence from citizen science monitoring. Insect Conserv. Divers..

[46] Wang L (2022). The relationship between green roofs and urban biodiversity: A systematic review. Biodivers. Conserv..

[47] Vergnes A, Viol IL, Clergeau P (2012). Green corridors in urban landscapes affect the arthropod communities of domestic gardens. Biol. Conserv..

[48] Blackhurst M, Hendrickson C, Matthews HS (2010). Cost-effectiveness of green roofs. J. Archit. Eng..

[49] Konvicka M, Kadlec T (2011). How to increase the value of urban areas for butterfly conservation? A lesson from Prague nature reserves and parks. Eur. J. Entomol..

[50] Krauss J, Alfert T, Steffan-Dewenter I (2009). Habitat area but not habitat age determines wild bee richness in limestone quarries. J. Appl. Ecol..

[51] Saarinen K, Valtonen A, Jantunen J, Saarnio S (2005). Butterflies and diurnal moths along road verges: Does road type affect diversity and abundance?. Biol. Conserv..

[52] Hool KD (2006). The effect of coal surface mine reclamation on diurnal lepidopteran conservation. J. Appl. Ecol..

[53] Tarrant S, Ollerton J, Rahman ML, Tarrant J, McCollin D (2012). Grassland restoration on landfill sites in the East Midlands, United Kingdom: an evaluation of floral resources and pollinating insects. Restor. Ecol..

[54] Heneberg P, Bogusch P, Řehounek J (2012). Sandpits provide critical refuge for bees and wasps (Hymenoptera: Apocrita). J. Insect Conserv..

[55] Lenda M, Skórka P, Moroń D, Rosin ZM, Tryjanowski P (2012). The importance of the gravel excavation industry for the conservation of grassland butterflies. Biol. Conserv..

[56] Moroń D (2014). Railway embankments as new habitat for pollinators in an agricultural landscape. PLoS ONE.

[57] Moroń D (2017). Do levees support diversity and affect spatial turnover of communities in plant-herbivore systems in an urban landscape?. Ecol. Eng..

[58] Tropek R (2012). Technical reclamations are wasting the conservation potential of post-mining sites. A case study of black coal spoil dumps. Ecol. Eng..

[59] Mata L (2020). Bringing nature back into cities. People Nat..

[60] Konvicka M, Fric Z, Benes J (2006). Butterfly extinctions in European states: do socioeconomic conditions matter more than physical geography?. Glob. Ecol. Biogeogr..

[61] Szala K, Kubicka AM, Sparks TH, Tryjanowski P (2020). Birds using tram tracks in Poznań (Poland): Species, infrastructure use and behaviour. Transp. Res. Part D Transp. Environ..

[62] Wiłkomirski B, Galera H, Sudnik-Wójcikowska B, Staszewski T, Malawska M (2012). Railway tracks—Habitat conditions, contamination, floristic settlement—A review. Environ. Nat. Resour. Res..

[63] Felcyn J, Preis A, Kokowski P, Gałuszka M (2018). A comparison of noise mapping data and people’s assessment of annoyance: How can noise action plans be improved?. Transp. Res. Part D Transp. Environ..

[64] Ministerstwo Rolnictwa i Rozwoju Wsi. *Etykiety, zezwolenia, pozwolenia i decyzje środków ochrony roślin*. (2023).

[65] Battisti L, Potrich M, Lozano ER, dos Reis Martinez CB, Sofia SH (2023). Review on the sublethal effects of pure and formulated glyphosate on bees: Emphasis on social bees. J. Appl. Entomol..

[66] Roberts, B. & Phillips, B. *Road verges and their potential for pollinators. A review of the costs, benefits and management options*. (Buglife, 2019).

[67] Gobel S (2006). Zunehmendes Interesse für die Tram ohne Fahrleitung. Stadtverkher.

[68] Wortmann, I. Deutschland mobil 2030—Zeit für neues Denken und Handeln. In *Mobilität der Zukunft* 97–102 (Springer Berlin Heidelberg, 2021).

[69] Guerrieri M (2019). Catenary-free tramway systems: Functional and cost-benefit analysis for a metropolitan area. Urban Rail Trans..

[70] Zhang W, Li J, Xu L, Ouyang M (2017). Optimization for a fuel cell/battery/capacity tram with equivalent consumption minimization strategy. Energy Convers. Manag..

[71] Pechanec V (2017). Monetary valuation of natural forest habitats in protected areas. Forests.

[72] Seják J, Pokorný J, Seeley K (2018). Achieving sustainable valuations of biotopes and ecosystem services. Sustainability.

